# Does genetic rescue disrupt local adaptation? An experimental test using thermally adapted *Tribolium castaneum* lines

**DOI:** 10.1098/rspb.2025.2036

**Published:** 2025-11-12

**Authors:** George West, Michael D Pointer, Will J Nash, Rebecca Lewis, David S Richardson

**Affiliations:** ^1^ School of Biological Sciences, University of East Anglia, Norwich, Norfolk, UK

**Keywords:** genetic rescue, *Tribolium castaneum*, inbreeding depression, local adaptation, thermal adaptation, outbreeding depression

## Abstract

Anthropogenic drivers are restricting many species to small, genetically isolated populations. These are prone to inbreeding depression and are at an increased risk of extinction. Genetic rescue, the controlled introduction of genetic variation from another population, can alleviate inbreeding effects. A major conservation concern restricting the use of this technique is that such augmented gene flow may disrupt local adaptation crucial to a population’s persistence. Using populations of the red flour beetle (*Tribolium castaneum*) experimentally adapted to reproduce at higher temperatures, we assess whether genetic rescue attempts disrupt thermal adaptation. Rescuers, drawn from populations adapted to either 30°C or 38°C, were introduced into populations adapted to 38°C, which had been inbred for two generations. We recorded population productivity for three generations post-rescue, in the adapted 38°C environment. Rescuers with and without local adaptation significantly increased the productivity of recipient inbred populations, but those sharing local adaptation to reproduction at 38°C provided greater increases in productivity. These results show that while rescue with non-locally adapted individuals may improve productivity, having the same adaptation in rescuing individuals and rescuee populations may be important in maximizing conservation outcomes.

## 1. Introduction

Climate change and habitat destruction are fragmenting species into increasingly small and isolated populations where gene flow is disrupted. This results in inbreeding, inbreeding depression and, consequently, increased risk of extinction [1–3]. Inbred individuals are more likely to be homozygous for any recessive, deleterious alleles present in the population, exposing their harmful effects [4,5]. This conversion of hidden genetic load, masked by dominance, into expressed load contributes to reduced fitness at both the individual and population levels. This increase in homozygosity may also lead such individuals to lose the benefits of heterozygote advantage [6]. Factors such as these interact with environmental drivers to push populations towards extinction [7–9].

Genetic rescue refers to the increase in fitness observed in an inbred population when novel genetic variation is introduced by a conspecific from another population, a ‘rescuer’ [10,11]. This process increases genome-wide heterozygosity within the target population, reducing expressed genetic load and improving fitness. Genetic rescue has been successfully implemented in endangered populations [12–15], including the Florida panther (*Puma concolor couguar*) [16,17]. Experimental organisms have also extended our understanding of genetic rescue [18–22], including how genetic rescue can affect adaptation in changing environments [23]. Despite this body of evidence supporting its efficacy, genetic rescue remains controversial among conservation managers [24–26].

If populations in need of genetic rescue exhibit local adaptation, the input of novel genetic variation from other populations could disrupt beneficial gene complexes. Rescuers may introduce non-locally adapted alleles, the expression of which could disrupt adaptation and thus exacerbate reduced fitness in vulnerable recipient populations [27,28]. Additionally, deleterious alleles from the rescuer population can be introduced, lowering fitness and potentially leading to population crashes [29,30]. This effect, termed outbreeding depression, has been suggested to be a key risk of implementing genetic rescue as a conservation measure [31,32]. However, it has been argued that, if genetic rescue guidelines are followed, the risk of such a detrimental impact is overstated [24,33,34], relative to the potential benefits of rescue. Indeed, while there are various examples of outbreeding depression occurring, the general consensus is that in many situations the benefits of reducing inbreeding depression outweigh the risk of outbreeding depression [35–38].

Selecting the source population for genetic rescue attempts is key to avoiding outbreeding depression. Populations from across a species range may be locally adapted to different conditions, leading to the introduction of maladaptive traits reducing population fitness [39]. Increasingly, reintroductions from captivity are being considered to reinforce wild populations including in the context of genetic rescue [40]. Captivity, however, could promote maladaptation as natural selection is weakened if not absent [41], utilizing individuals from captivity for rescue could reduce the fitness of wild populations by introducing maladapted genotypes. Testing the effects of maladaptation on genetic rescue, from wild or captive populations, is vital to improve our ability to select the best source population for rescuers.

Climate change poses a significant challenge for endangered species [42], with genetically depauperate populations struggling to adapt [43]. The introduction of genetic variation into isolated populations via genetic rescue could expedite adaptation to rapidly evolving climate by supplementing standing genetic variation [32,44]. Seeding rescue attempts from populations with specific local adaptations, for example to high temperatures [45], could facilitate the introgression of beneficial adaptations into endangered populations [46,47]. Genetic rescue of inbred endangered populations that exhibit specific local adaptations is also a key consideration to protect such unique combinations of alleles to support future species-level resiliency.

*Tribolium castaneum*, a tenebrionid beetle, is a model organism [48] for population genetics, genetic rescue and thermal tolerance [18,49,50]. Here, we use *T. castaneum* populations experimentally selected for over 150 generations to reproduce at 38°C, compared to the ancestral population optimum of 30°C [51,52]. The thermally adapted populations maintain fecundity when developing at 38°C; they produce more eggs and have a greater hatching success than non-adapted populations developing at this temperature [53]. However, these thermally adapted populations produce fewer offspring than do non-adapted populations that have developed at 30°C, even when those non-adapted populations are transferred to mate and oviposit at 38°C as adults [53]. This suggests that adaptation to a higher temperature may carry a fitness cost. In order to test the impact of genetic rescue from rescuing individuals with and without local adaptations, we used replicated subpopulations of these adapted lines to generate inbred experimental populations. We assessed the impact of genetic rescue from individuals with different adaptive backgrounds on thermal tolerance adaptation. We show that genetic rescue by an adapted individual was the most effective treatment at increasing fitness in inbred populations.

## 2. Methods

### (a) Husbandry

*Tribolium castaneum* populations were maintained on standard fodder (90% white organic flour, 10% brewer’s yeast and a layer of oats for traction) in a controlled environment of 30°C (unless otherwise stated) and 60% humidity with a 12:12 light:dark cycle. Populations were maintained following a standard cycle of virgin adults having 7 days of mating and oviposition followed by the removal of adult beetles, using 2 mm and 850 µm sieves, so that only eggs remain in the fodder. Each generation is initiated with a number of adults (line dependent, see below) that are given 7 days to mate and lay eggs before being removed. The eggs are left for 35 days to develop into mature adults.

### (b) *Tribolium castaneum* lines

#### (i) Krakow super strain

Krakow super strain (KSS) is a combination of 14 laboratory strains that were bred to maximize genetic diversity in one population maintained at a census size of 600 individuals [54]. This line is highly productive at 30°C but has reduced fitness when developing at 38°C. This line is highly productive at 30°C but has reduced fitness when developing at 38°C. The number of eggs produced by a single KSS female developing at 38°C over 2 days declines from ~22 to ~11 and the proportion of eggs that hatch falls from ~40% to nearly 0% [53]. This line was used as the non-adapted rescuer population.

#### (ii) Thermal lines

Ten independent lines (census size = 100 adults) were founded from KSS and experimentally evolved for ~150 generations at an environmental temperature of 38°C [55]. This imposed selection for development and reproduction at this temperature, considerably above the thermal optimum for *T. castaneum* [56]. All other conditions were as described above aside from a shorter development period of 27 days, reflecting accelerated development at 38°C. A single thermally adapted female can produce more eggs over 2 days than KSS when developing at 38°C, around 18 eggs with around a 50% hatching proportion [53]. These were used as the thermally adapted rescuer populations.

#### (iii) Inbred lines

Ten inbred thermally adapted populations were created; each descended from one of the ten thermal lines described above. Adult beetles from each thermal line were housed individually for two weeks to ensure any fertilized eggs were laid. Single-pair matings were formed by housing together a previously isolated male and a female for 7 days of mating and oviposition, resulting in a single pair bottleneck for each thermal line. Full-sibling offspring resulting from this pairing were again paired for a second bottleneck. The following generation was initiated with 10 male and 10 female full-sibling offspring of full-sibling pairs. From the offspring of these groups, 6 inbred experimental populations (10 males and 10 females) were created from each of the 10 inbred thermal lines, to act as recipient populations for genetic rescue. One inbred population only produced 4 experimental populations, resulting in a total of 58 experimental populations split over two temporal blocks of 30 and 28. The two blocks were maintained one day apart for ease of handling but otherwise received identical treatment. Each population received a random ID number to blind the experiment and avoid bias. Experimental populations were initiated every generation using 10 males and 10 females sourced from the offspring of the previous generation to reduce density-dependent effects [57–59]. All experimental inbred recipient populations were kept at 38°C in A. B. Newlife 75 Mk4 forced air egg incubators (A. B. Incubators, Suffolk, UK); all other conditions were kept as described above.

### (c) Genetic rescue protocol

Populations were kept in 125 ml tubs containing 70 ml of standard fodder. After 7 days, adults were discarded, and eggs were left to develop for ~21 days when 10 male and 10 female pupae were randomly selected to establish the next generation. Remaining individuals were maintained for 10 days following this and were then frozen and manually counted. Pupae taken at day ~21 were housed in plastic dishes containing 10 ml standard fodder in single-sex groups until they matured into adults after 10 ± 2 days, and the next generational cycle began with unmated adults, avoiding overlapping generations.

**Figure 1.**
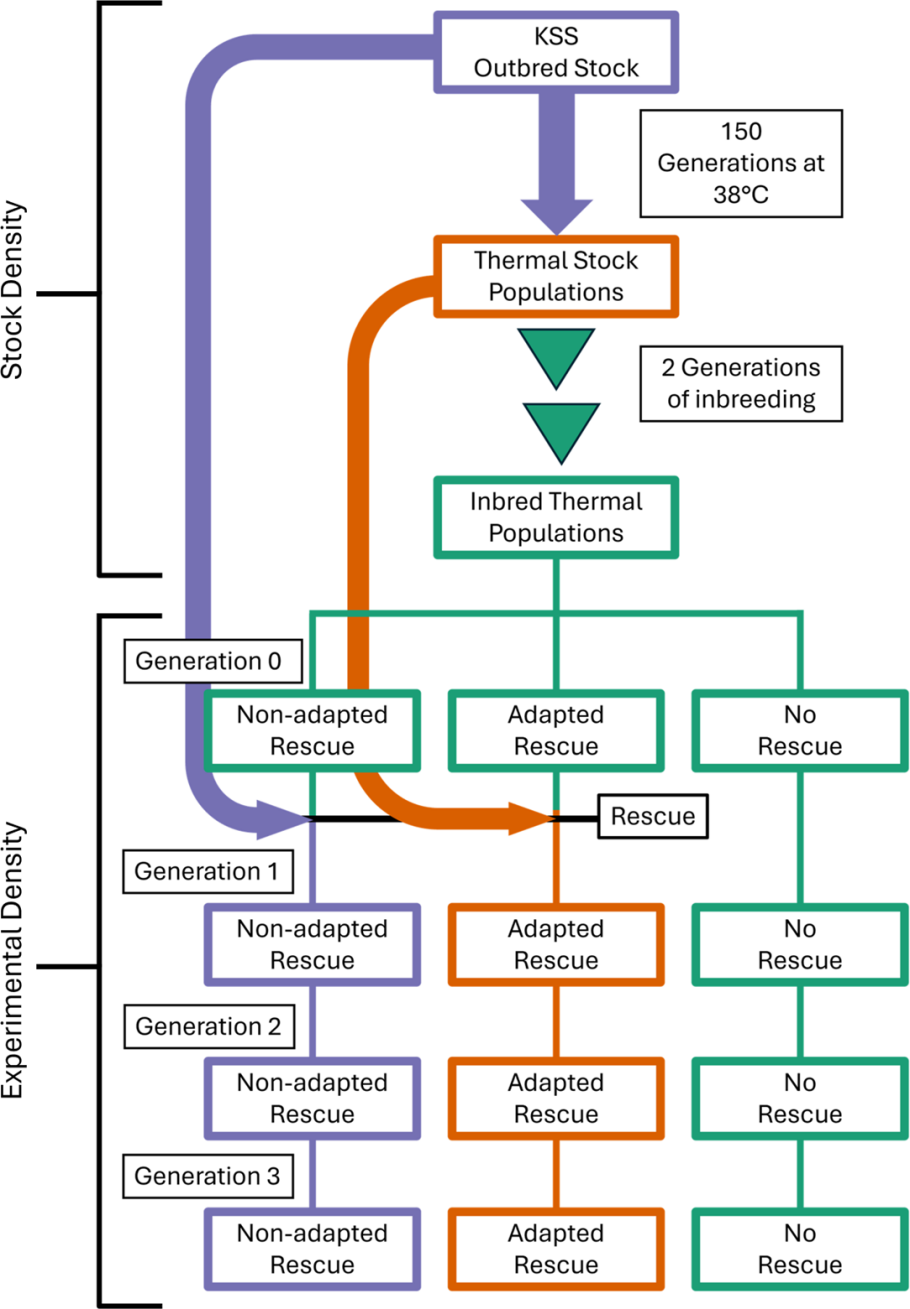
Experimental design for the attempted genetic rescue of inbred thermally adapted *T. castaneum* populations by a single thermally adapted or non-thermally adapted rescuer. Inbred, thermally adapted lines were created by inbreeding lines thermally adapted to 38°C over ~150 generations [55] with two generations of full-sibling matings, before being kept for three generations at *n* = 20 (10 females and 10 males) during the experiment. The 10 inbred thermal lines were replicated to be represented in each experimental treatment twice. The final sample size was 56 experimental populations (see main text).

After a rest generation a single male from each inbred recipient population was removed and replaced with a single male rescuer to avoid demographic rescue effects (increased population fitness due to increased population size) [1,10,32]. Three treatments were created: (i) control—19 populations received no rescue (the male was not removed); (ii) locally adapted rescue—19 populations received a 38°C-adapted rescuer (a male from a different thermally adapted population); (iii) non-locally adapted rescue—20 populations received a non-thermally adapted rescuer (a KSS male, see above) (figure 1).

Population fitness was measured using productivity: the number of mature adult offspring the population produced each generation [60]. Populations were maintained for three non-overlapping generations following rescue. Two replicates were lost after two generations due to human error, resulting in a third generation with 19 control, 18 thermally adapted rescue and 19 non-thermally adapted rescue populations. Data for populations lost in generation 3 were included in the analysis of generations 1 and 2.

### (d) Statistical analysis

R v.4.4.1 [61] was used with R Studio version 2024.04.2 + 764 [62]. Data management and exploration were performed with tidyverse [63], stats [61], Rmisc [64] and googlesheets4 [65]. ggplot2 [66] was used to visualize results. Data distribution was checked using the shapiro.test function [61]. The glmmTMB package [67] was used to fit generalized linear mixed models (GLMMs). DHARMa [68] was used to check model fit and the check_collinearity function from the performance package [69] to test variance inflation factor (VIF) scores. No overdispersion or collinearity (VIF < 3 for all variables) was found. *R*^2^ was determined using the r.squaredGLMM function in MuMIn [70].

Counts of population productivity over all generations were analysed using a GLMM with a negative binomial errors and a log link function. Fixed explanatory variables were rescue treatment and generation as well as the interaction between these variables. To account for population variance and relatedness between replicate populations, a random factor was added, nesting individual ID within the stock thermal line from which the population descended. The control treatment was set as the baseline factor for comparison. The baseline was changed to non-adapted to compare between the two rescue treatments post-hoc.

GLMMs, constructed as described above, but excluding the generation variable, were then run post-hoc on each generation individually to test if there were significant differences between the treatments in each generation. The baseline was changed to non-adapted rescuers, to compare the two rescue treatments post-hoc.

## 3. Results

**Figure 2.**
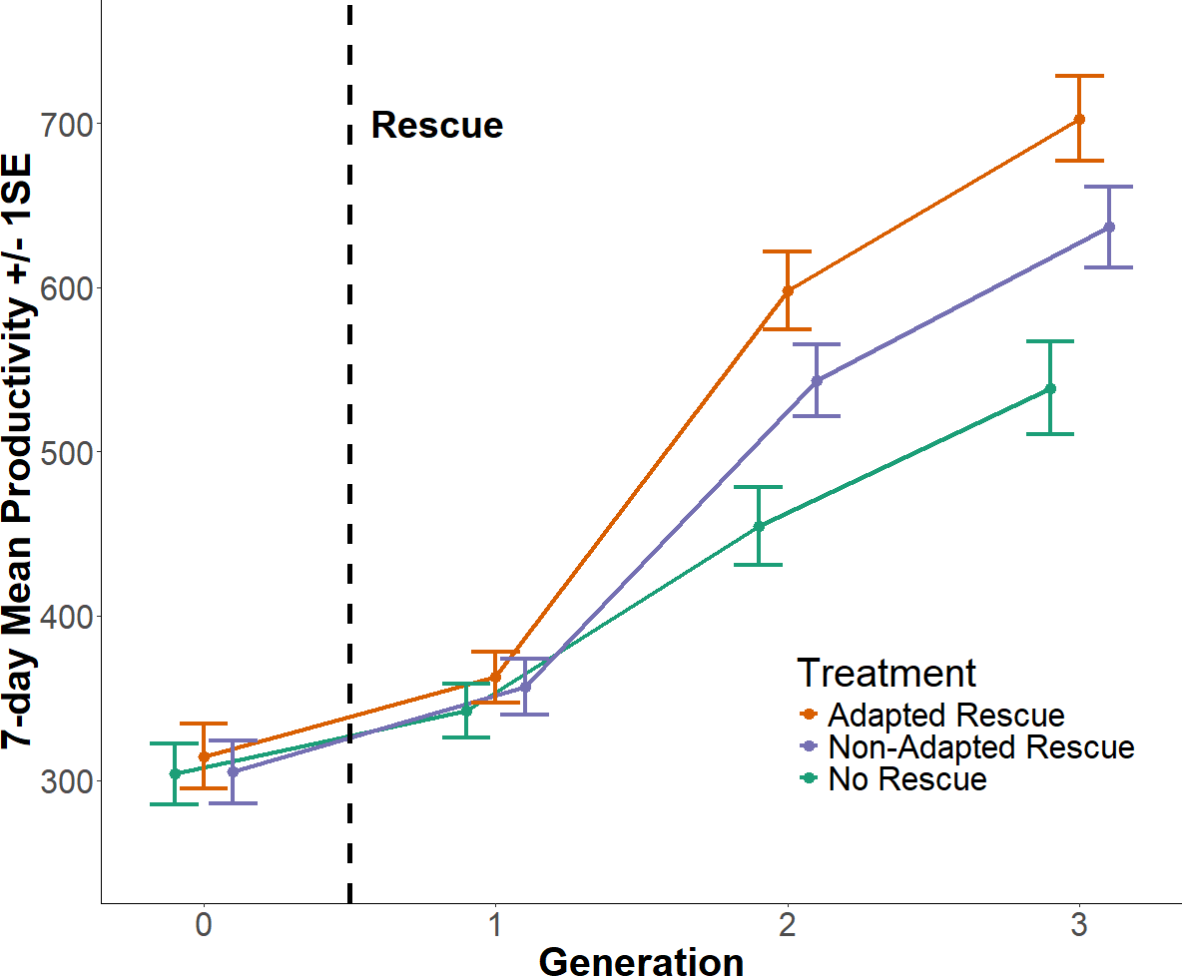
The effect of introducing a (1) thermally adapted (orange) or (2) non-adapted (blue) rescuer, compared to control populations (green) on the mean productivity of inbred thermally adapted populations of *T. castaneum* over three generations after a single introduction event (dotted vertical line) while maintained at 38°C (population size = 20; number of experimental populations = 58 in generations 1 and 2, 56 in generation 3). Generation 0 was not included in statistical analysis. Plot is jittered to aid in visualization, error bars represent ± 1 s.e.

The interaction between treatment and generation was significant for the thermally adapted rescue (*β* = 0.093 ± 0.039, *z* = 3.38, *p* = 0.017) but not for the standard rescue (*β* = 0.055 ± 0.039, *z* = 1.41, *p* = 0.16) when each was compared with the no-rescue control (table 1, figure 2). A post-hoc contrast between the two rescue treatments showed no difference in the gradient of productivity change over generation (*β* = 0.038 ± 0.037, *z* = 1.01, *p* = 0.31, 95 % CI = –0.035 to 0.111).

**Table 1.** Summary of a GLMM fitted to model the productivity of small, inbred, thermally adapted *T. castaneum* populations (population size = 20, number of populations = 58 or 56) after receiving a rescue by a thermally adapted, or non-adapted, male rescuer or no rescue over three generations. Predictors in bold are significant (*p* < 0.05). Marginal *R*^2^ = 0.637, conditional *R*^2^ = 0.740. The treatment (no rescue) was the baseline for comparison in the model, the values in the table are relative to that treatment.

predictor	estimate	s.e.	*z*	*p*
intercept	5.626	0.0756	47.43	<2e−16
treatment (no rescue)				
thermally adapted rescue	0.024	0.096	0.25	0.803
non-adapted rescue	0.028	0.096	0.29	0.771
**generation**	**0.224**	**0.031**	**7.33**	**<0.001**
treatment (no rescue) × generation				
**treatment (thermally adapted rescue) × generation**	**0.091**	**0.041**	**2.21**	**0.027**
treatment (non-adapted rescue) × generation	0.055	0.041	1.33	0.185
random	172 observations		variance	
ID:thermal line	58		0.002	
thermal line	10		0.007	

In generation 1, no significant differences existed between any of the treatments (non-adapted—adapted: estimate = 0.015, s.e. = 0.057, *z* = 0.26, *p* = 0.794; table 2). In generations 2 and 3, productivity in the rescue treatments differed significantly from the no rescue control, were not significantly different in generation 2 (estimate = 0.095, s.e. = 0.057, *z* = 1.67, *p* = 0.095), but exhibited a significantly difference in generation 3 (estimate = 0.097, s.e. = 0.045, *z* = 2.17, *p* = 0.030).

**Table 2.** Composite table of three GLMM results for each generation of the productivity of small, inbred, thermally adapted *T. castaneum* populations (population size = 20, experimental populations = 58 or 56) after receiving a rescue by a thermally adapted, or non-adapted, male rescuer or no rescue. Predictors in bold are significant (*p* < 0.05).

	gen 1 estimate	s.e.	*z*	*p*	gen 2 estimate	s.e.	*z*	*p*	gen 3 estimate	s.e.	*z*	*p*
intercept (no rescue)	5.810	0.055	105.84	<2e-16	6.100	0.057	107.29	<2e-16	6.263	0.051	122.93	<2e-16
thermally adapted rescue	0.061	0.058	1.05	0.291	0.284	0.058	4.90	**<0.001**	0.277	0.045	6.20	**<0.001**
non-adapted rescue	0.056	0.057	0.81	0.420	0.188	0.057	3.28	**0.001**	0.180	0.044	4.07	**<0.001**
	gen 1 observation		gen 1 variance		gen 2 observation		gen 2 variance		gen 3 observation		gen 3 variance	
random	58				58				56			
ID:thermal	58		<0.001		58		0.030		56		0.017	
thermal	10		<0.001		10		0.005		10		0.016	

## 4. Discussion

We tested the disruptive effect of genetic rescue in locally adapted populations, by introducing thermally adapted or outbred, non-adapted genetic rescuers to inbred thermally adapted populations of *T. castaneum* and then measuring productivity as an estimate of population fitness. We show that populations receiving thermally adapted rescue recovered fitness following two generations of inbreeding at a faster rate than populations that did not receive new genetic variation. When looking at each generation individually, both rescue treatments improved fitness in the second and third generations, when compared to the no-rescue control. In the third generation, fitness in populations that received thermally adapted rescue improved over both the no-rescue control and non-adapted rescue treatment. We also observed increased productivity in the no rescue treatment, this was seen in previous experiments [71] and is probably due to the change from highly dense stock populations to low density experimental populations effecting all populations equally [57]. This makes the inclusion of no rescue control populations vital, comparisons before and after rescue would be misleading without the data they provide.

Introducing new genetic diversity into an inbred population is predicted to improve population fitness, and such genetic rescue effects have been observed in applied, unreplicated conservation studies [72,73]. Here, we provide experimental support for this suggestion, as well as showing that the fitness of locally adapted, inbred populations recovered at the greatest rate following the introduction of a rescuing individual from a population with a similar selective background. Importantly, we show that non-adapted rescuers also improved fitness compared with no rescue, despite the potential to disrupt adaptation, though the magnitude of the fitness increase was smaller than when using a locally adapted rescuer. The benefits of rescue were also clear even utilizing only a single rescuer in a single rescue event in the experiment, which has also been observed in *Drosophila* [74], supporting the one-migrant-per-generation rule for preventing inbreeding depression [75]. Recent experimental studies have also shown that the benefits of rescue can be long-lasting, far beyond the three generations followed here [22].

The ancestral population in our experiment is highly outbred and should represent an ideal source of variation for use in genetic rescue [33]. Our findings show that local adaptations, evolved over 150 generations, considerably increased the efficacy of genetic rescue. Previous studies have suggested that the reinitiation of gene flow between populations following more than 20 generations of environmental divergence may risk outbreeding depression [24]. However, there is a growing body of evidence that the risk of outbreeding depression in genetic rescue may be exaggerated [34,76,77]. In our study, the relatedness of the lines, benign conditions and experimental set-up make outbreeding depression unlikely to occur, excepting the disruption of adaptation to increased temperature.

In our study, genetic rescue by a single individual bearing local thermal adaptation was more effective than rescue by an outbred rescuer not bearing this adaptation. Our findings support recommendations that genetic rescue should utilize source populations inhabiting similar environments, reducing the risk of disrupting local adaptation [28]. We used 10 independent thermally adapted lines in this study, all originating from the same genetically diverse ancestral population. As these populations adapted to 38°C independently [51], they may represent differing subsets of genetic variation, providing alternative substrates for genetic rescue to act on. This design provides a proxy for population fragmentation, with subpopulations diverging from a larger outbred population. As the experimental evolution for thermal adaptation probably resulted in bottlenecking of the adapting populations [55], we predict these populations to be less genetically diverse than the outbred ancestral population.

In the thermally adapted populations we study, the key adaptation is the capacity to remain fertile while developing at 38°C [51,53,78]. Our findings suggest that the introduction of outbred variation may have disrupted this adaptation. If the increase in fitness were solely attributable to the introduction of genetic variation, an outbred rescuer would generate the most impactive rescue effect [33]. We show that the addition of locally adapted variation was more important to rapid recovery from inbreeding effects, supporting the importance of rescue from populations with similar adaptive backgrounds [25]. Using translocations from locally adapted populations could be important in a conservation context as they may improve population resilience to a changing climate [76].

It is beyond the scope of this study to identify the causative genetic variation that conferred local adaptation and that allowed locally adapted individuals to act as the most effective rescuers. Future work should aim to test if effective rescue is mediated by the transfer of beneficial alleles directly contributing to adapted traits [46], or by the purging of genetic load, mildly deleterious mutations, in genes associated with the adaptation [79]. Despite this lack of resolution, we use a powerful, highly replicated, experimental design to answer a question difficult to test in wild populations. Our findings are highly relevant to conservation scenarios and help highlight the utility of experimental evolution in contributing to applied questions. We provide support for the consideration of local adaptation in genetic rescue programmes showing that single rescuer can provide clear fitness benefits and adapted populations are a better source of rescuers.

## Data Availability

All data and scripts are available at Dryad [60]. Supplementary material is available online [80].
